# Effect of soy isoflavones on the growth of human breast tumors: findings from preclinical studies

**DOI:** 10.1002/fsn3.142

**Published:** 2014-07-10

**Authors:** Youngjoo Kwon

**Affiliations:** Department of Food Science and Engineering, Ewha Womans UniversitySeoul, Korea

**Keywords:** Breast cancer, isoflavones, soy

## Abstract

Breast cancer is the most common cancer among women worldwide, and many women with breast cancer live more than 5 years after their diagnosis. Breast cancer patients and survivors have a greater interest in taking soy foods and isoflavone supplements. However, the effect of isoflavones on breast cancer remains controversial. Thus, it is critical to determine if and when isoflavones are beneficial or detrimental to breast cancer patients. According to the available preclinical data, high concentrations of isoflavones inhibit the proliferation of breast cancer cells, regardless of their estrogen receptor (ER) status. In comparison, genistein, a major isoflavone, has stimulated tumor growth at low concentrations and mitigated tamoxifen efficacy in ER-positive breast cancer. Studies have indicated that the relative levels of genistein and estrogen at the target site are important to determine the genistein effect on the ER-positive tumor growth. However, studies using ovariectomized mice and subcutaneous xenograft models might not truly reflect estrogen concentrations in human breast tumors. Moreover, it may be an oversimplification that isoflavones stimulate hormone-dependent tumor growth due to their potential estrogenic effect since studies also suggest nonestrogenic anticancer effects of isoflavones and ER-independent anticancer activity of tamoxifen. Therefore, the concentrations of isoflavones and estrogen in human breast tumors should be considered better in future preclinical studies and the parameters that can estimate those levels in breast tumors are required in human clinical/epidemiological investigation. In addition, it will be important to identify the molecular mechanisms that either inhibit or promote the growth of breast cancer cells by soy isoflavones, and use those molecules to evaluate the relevance of the preclinical findings to the human disease and to predict the health effects of isoflavones in human breast tumors.

## Introduction

The incidence of cancer increases globally. In GLOBOCAN 2012 estimates, breast cancer has attracted more attention due to its marked increase in both incidence (over 20%) and mortality (14%) worldwide, compared to 2008 estimates (Ferlay et al. 2013). In 2012, 1.7 million women were diagnosed with breast cancer, making it the most frequently diagnosed cancer among women in 140 out of 184 countries (Ferlay et al. 2013). In addition, 6.3 million women are estimated to have lived with breast cancer (diagnosed within 5 years) (Ferlay et al. 2013). Therefore, besides breast cancer prevention efforts, better healthcare procedures for breast cancer patients and survivors are in high demand.

Cancer patients and survivors present greater interest in their food choices and the use of dietary supplements (Velentzis et al. 2011). The majority of people diagnosed with cancer use (or consider using) some form of dietary supplements to relieve the toxic effects of chemotherapy or increase the efficacy of their therapeutic treatments (Boon et al. 2000; Block et al. 2008). Soy, available in dietary supplements, is often considered by women at high risk for breast cancer (Fang et al. 2005). Many women who have breast cancer risk factors increase their soy intake, assuming that high consumption manifests an anticancer effect (Fang et al. 2005).

Soy has been consumed in high quantities in Asia where breast cancer incidence is relatively lower than in Western countries; and thus, the high consumption of soy food has been considered to prevent the development of breast cancer (Miller 1977; Gray et al. 1979; Lee et al. 1991). Many studies have demonstrated the breast cancer prevention effects of soy isoflavones, such as genistein and daidzein, the most recognized biologically active compounds in soy (Barnes et al. 1995; Kennedy 1995). It is generally considered that the high consumption of soy foods, particularly during childhood and early adolescence, helps mammary gland development and breast tissue differentiation, thereby contributing to protection from breast cancer development (Lamartiniere 2000; Duffy et al. 2007; Dewi et al. 2013). However, it remains controversial whether soy isoflavones are safe to be consumed by breast cancer patients or women at high risk for breast cancer. Isoflavones are major food-derived phytoestrogens that are structurally similar to estrogen. Thus, isoflavones can weakly bind to estrogen receptor (ERs) when the local estrogen concentration is low (Martin et al. 1978). This potential estrogenic effect of soy isoflavones raises the concern that the high consumption of soy foods by breast cancer patients or women at high risk for breast cancer may increase estrogen-dependent breast tumor growth (Hsieh et al. 1998).

Since the potential cancer promotion effect of isoflavones was reported (Hsieh et al. 1998; Allred et al. 2001a,b; Ju et al. 2001), many studies have been undertaken to determine the effect of soy isoflavones on breast cancer risk (Lamartiniere 2000; Fang et al. 2005; Duffy et al. 2007; Kang et al. 2010; Nechuta et al. 2012). However, it is still uncertain whether soy isoflavones present a risk factor for breast cancer. Subsequently, dietary guidelines for breast cancer patients and/or women at high risk are inconsistent (Duffy et al. 2007; Boucher et al. 2012). Recent reports have indicated that newly diagnosed breast cancer patients start or stop taking soy foods after the diagnosis although the right approach is unknown (Boucher et al. 2012). Phytoestrogens including soy isoflavones are also known to relieve menopausal symptoms such as hot flashes, decrease LDL cholesterol, and prevent osteoporosis, although more studies are required (Glazier and Bowman 2001; Farrell 2003). In addition, soy foods are a good source of essential amino acids, especially for people on vegetarian diets. Many women may either unnecessarily avoid soy foods or try to consume in a high quantity even when they should not. Thus, it is very important to establish whether soy or isoflavones can potentially increase breast cancer risk, and whether soy intake should be avoided in women at high risk for breast cancer and breast cancer patients.

In this article, preclinical studies that have evaluated the effect of soy isoflavones on the growth of breast cancer cells will be summarized. This will help derive the factors that cause the inconsistent health effects of soy isoflavones, leading the discussion on the considerations required in both preclinical and human clinical/epidemiological studies to help understand the effect of soy isoflavones on human breast tumor behavior.

## Effect of Genistein on the Growth of Breast Cancer Cells

It has been shown that soy isoflavones, predominantly genistein, exert biological activity. The suggested mechanisms of genistein action include estrogen agonist/antagonist activity (Martin et al. 1978), the inhibition of receptor tyrosine kinase (Akiyama et al. 1987; Dean et al. 1989), the inhibition of topoisomerase II (Okura et al. 1988; Markovits et al. 1989), and the induction of differentiation through the inhibition of protein phosphorylation and cell cycle arrest (Watanabe et al. 1989;  l, Rahal and Simmen 2010). The ER status of breast cancer has been an important factor in consideration of genistein effect on tumor growth.

### Effect on ER-positive breast cancer cells

Although many studies have been performed to determine the anticancer effects of genistein, genistein's effect on the growth of ER*α*-positive breast cancer is still equivocal. One of the factors that drive these inconsistent results seemed to be the difference in the genistein dose used in different studies. Relatively high concentrations of genistein (at a 50 or 100 *μ*mol/L level) inhibited the proliferation of human breast cancer cells, including ER-positive MCF-7 cell line (Peterson and Barnes 1991, 1996; Pagliacci et al. 1994; Chen et al. 2003). Daidzein, another major soy isoflavone, required more than twice this concentration of genistein in order to suppress breast cancer cell proliferation and isoflavone beta-glucosides (e.g., genistin and daidzin) had little effect on the cell growth (Peterson and Barnes 1991, 1996). The inhibition of cell proliferation by 50–100 *μ*mol/L of genistein did not involve the inhibition of tyrosine receptor kinases such as epidermal growth factor receptor (EGFR), and the inhibition of EGFR phosphorylation required even a higher concentration (about 370 *μ*mol/L) of genistein (Peterson and Barnes 1996). However, studies consistently demonstrated that genistein induced cell cycle arrest and apoptosis at concentrations of 50–100 *μ*mol/L (Pagliacci et al. 1994; Chen et al. 2003), although it is unclear whether ERs mediates genistein-induced growth inhibition. Some studies reported that the inhibition of cell growth by genistein at this high concentration did not necessarily depend on functional ERs (Peterson and Barnes 1996). In contrast, the study of Chen et al. (2003) suggested ER-dependence in the genistein-mediated growth inhibition. In response to 50 and 100 *μ*mol/L genistein exposure, MCF-7 cells down-regulated mRNA levels of*serum response factor* (*SRF*) and*ERα*, making cells arrest at the G_2_/M phase (Chen et al. 2003). Therefore, a high concentration of genistein (>25 *μ*mol/L) inhibits the growth of ER-positive breast cancer cells in vitro through both ER-dependent and -independent mechanisms.

Genistein oppositely enhanced the growth of hormone-dependent breast cancer cells at low concentrations, stimulating the growth of estrogen-dependent MCF-7 cells in vitro at the range of 0.01–10 *μ*mol/L, in contrast to the inhibition of MCF-7 cell growth at higher concentrations (>25 *μ*mol/L) (Martin et al. 1978; Hsieh et al. 1998). The growth stimulating effect of genistein was more apparent when estrogen was absent or negligible (i.e., when dextran-charcoal-stripped fetal bovine serum was used in the culture media) (Martin et al. 1978). Consistently, dietary genistein (250–1000 mg/kg, equivalent to 0.39–3.36 *μ*mol/L plasma concentrations) promoted the growth of tumors derived from MCF-7 cells that were subcutaneously injected in ovariectomized athymic mice (Hsieh et al. 1998; Allred et al. 2001a,b; Ju et al. 2001). The estrogenic effect of genistein has been implicated in the genistein-induced growth stimulation of MCF-7 cells (Ju et al. 2001). Thus, genistein at a physiologically achievable level promoted the growth of ER-positive breast tumor cells, probably due to the estrogen agonistic activity of genistein (Hsieh et al. 1998; Allred et al. 2001a,b; Ju et al. 2001).

Based on the studies above, less than 10 *μ*mol/L of genistein enhances the growth of breast cancer cells, whereas genistein suppresses cell proliferation at the concentration range of 50–100 *μ*mol/L. This leads us to the question whether this high concentration of genistein can be achieved in vivo. Dietary genistein at the dose of 6000 mg/kg resulted in less than 7 *μ*mol/L of genistein in the plasma of nude mice (Santell et al. 2000). Although subcutaneously administered genistein (0.5 mg/kg) suppressed the growth of tumors derived from MCF-7 cells in female nude mice (Shao et al. 1998), the subcutaneous delivery might lead to higher plasma concentrations than those achieved by oral intake. In this study, the plasma level of either genistein or estrogen was not estimated, making it difficult to interrelate this with in vitro results. In addition, bioavailable genistein would be 20–150 times lower in humans compared to rodents, due to the high level of isoflavone metabolism in humans (Setchell et al. 2011). Furthermore, the intake of soy-based food supplements for five consecutive days gave rise to 100-fold lesser concentrations of genistein, daidzein, and equol in breast tissues, compared to the serum of the same individual who underwent a mastectomy (Maubach et al. 2004). Therefore, it is not very likely that soy intake leads to a genistein concentration high enough to exert the growth-inhibitory effect on human breast tumors.

**Figure 1:**
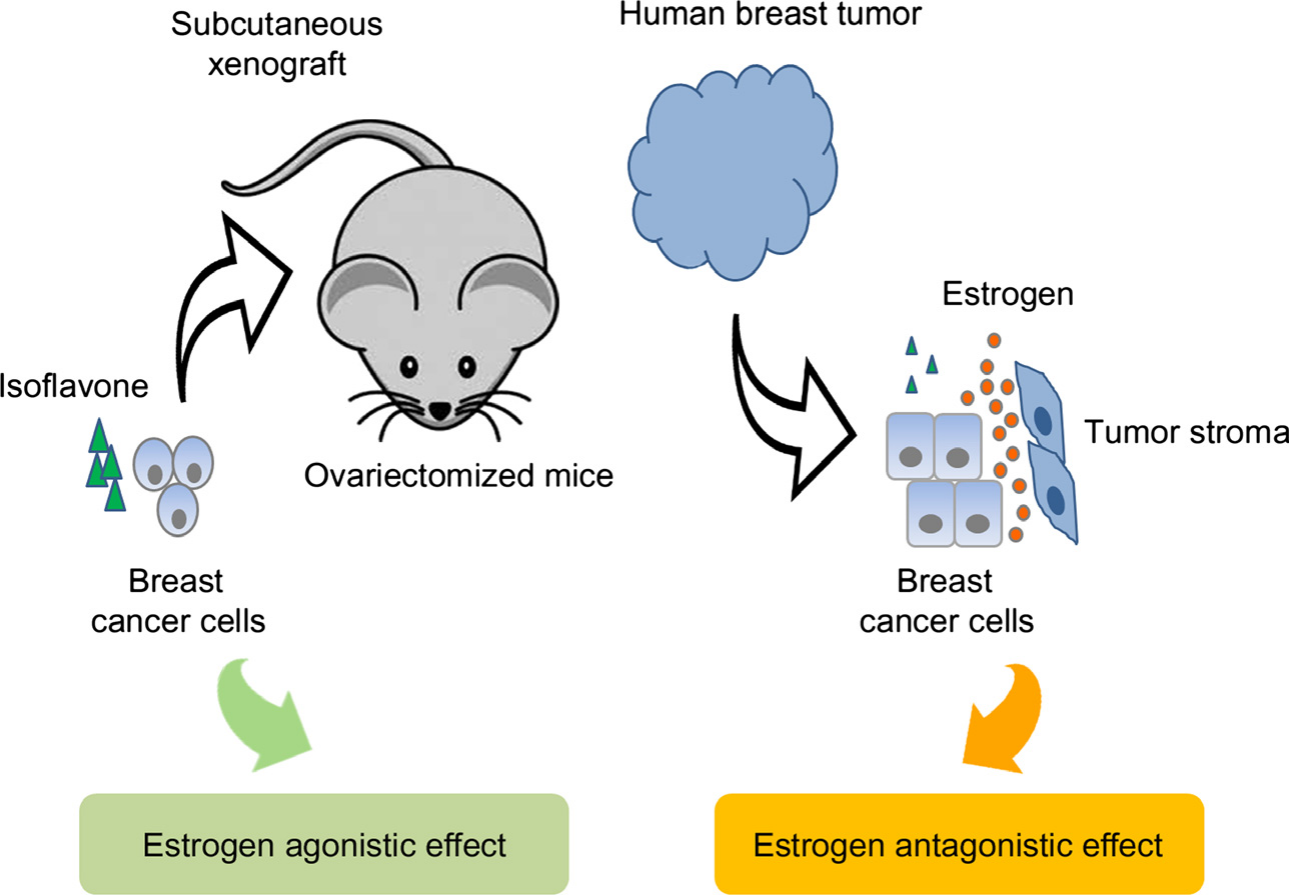
The relative level of isoflavones and estrogen at the target site is an important factor that determines potential estrogen agonistic/antagonistic effect of soy isoflavones on the ER-positive tumor growth. Preclinical studies that use subcutaneous xenografts models might not properly reflect the relative levels of estrogen and isoflavones on human breast tumor where estrogen is provided by the surrounding stroma and isoflavones are less bioavailable. In this circumstance, dietary isoflavones might not have a significant estrogen agonist effect and, rather, could exert an estrogen antagonistic effect. Therefore, the relative levels of isoflavones and estrogen on human breast tumors should be considered more carefully in the preclinical study design and parameters that can estimate those levels should be included to determine the effect of isoflavones on the growth of human breast tumors in clinical/epidemiological studies.

Another question is whether the estrogen concentration in the breast tissue has been appropriately considered. It was suggested that the relative level of genistein and estrogen is an important factor that determines potential estrogen agonistic/antagonistic effect of genistein and the consequence of genistein intake on the growth of ER-positive breast tumor. Accordingly, many studies have tested whether genistein may act as an estrogen agonist and promote the growth of breast tumor under low local concentrations of estrogen (e.g., in postmenopausal women) (Hsieh et al. 1998; Allred et al. 2001a,b; Ju et al. 2001). In fact, genistein presented a greater growth stimulatory effect under the condition where estrogen was removed as much as possible (Martin et al. 1978). However, it should be also noted that estrogen levels in normal breast tissues are high and breast tumors have 10–50-fold higher estrogen level compared to the level in the blood, even in postmenopausal women (Yaghjyan and Colditz 2011). Moreover, it has been demonstrated that stromal cells surrounding breast tumors are the primary source of estrogen (Santen et al. 1997). In comparison, studies that presented the growth stimulatory effect of dietary genistein used a subcutaneous xenografts model (inoculating cancer cells into the flank of mice) in ovariectomized mice without providing extraneous estrogen. The flank of ovariectomized mice might present conditions of estrogen deprivation; therefore, the study design might not properly reflect the estrogen concentration in the breast tumor microenvironment. In the human breast tumor, the estrogen concentration might be higher whereas the isoflavone concentration might be lower (Maubach et al. 2004; Setchell et al. 2011) compared to the levels considered in the preclinical studies (Fig.1). In this circumstance, dietary genistein might not have a significant estrogen agonistic effect and, rather, could exert an estrogen antagonistic effect (Fig.1). It will be more informative to utilize culture systems or orthotopic animal models that consider the primary site of tumor formation as well as interactions of tumor cells with their physiological microenvironment (Killion et al. 1998; Cordero et al. 2010) to better reflect estrogen level at the target sites. It will be also important to include parameters that estimate the relative levels of estrogen and genistein concentrations in human breast tumors in clinical and epidemiological studies.

MCF-7 cells were almost exclusively used as an estrogen-dependent ER*α*-positive breast cancer model. It is unknown whether certain genetic or phenotypic characteristics related to the MCF-7 cell line caused the genistein effect on cell proliferation. Different cells had different susceptibilities to genistein-induced growth inhibition, even within the same ER status (Peterson and Barnes 1996). Therefore, it would be desirable to include other ER*α*-positive breast cancer cells or primary cells derived from ER-positive human breast tumors to generalize the findings of genistein effect on the MCF-7 cell growth. In addition, there is limited information on molecular mechanisms how genistein promotes or inhibits the proliferation of cancer. Nanomolar concentrations of genistein stimulated MCF-7 cell proliferation by inducing transcriptional elevation of the acid ceramidase gene,*ASAH1*, in vitro (Lucki and Sewer 2011). However, this result has not been confirmed in either other cell lines or animal models. More detailed molecular mechanisms than the estrogen agonist effect will provide a better rationale for the inhibition of soy intake in breast cancer patients or women at high risk for breast cancer. Identified molecules can be also utilized as markers that monitor the response to genistein intake and predict human tumor behaviors, evaluating the relevance of preclinical results to the human disease. Therefore, it will be necessary to identify the molecular mechanisms by which genistein inhibits or enhances proliferation of ER-positive breast cancer cells.

### Effect on ER-negative breast cancer cells

Genistein's effect on hormone-independent breast cancer cells has been less studied. Genistein did not present any effect on hormone-independent breast cancer cells, such as MDA-MB-231 cells, at low concentrations (<10 *μ*mol/L) and inhibited cellular proliferation only at relatively high concentrations (>20 *μ*mol/L) in vitro (Santell et al. 2000). Dietary genistein (750 mg/kg) did not inhibit the growth of MDA-MB-231 tumor xenografts in vitro, probably because of low bioavailable concentration of genistein (<7 *μ*mol/L plasma concentration) (Santell et al. 2000). However, another study reported that a lower dose of dietary genistein (250 mg/kg) inhibited the growth of MDA-MB-231 cells orthotopically implanted into female nude mice (Li et al. 2013). The latter study used an orthotopic mouse model, which considers human physiological conditions better compared to conventional subcutaneous implantation of tumor cells, as conducted by Santell et al. Another major difference between the two studies is the timing of dietary exposure to genistein. Genistein was provided prior to cell inoculation in Li et al.'s study, whereas tumor was first developed (for about 5 weeks) before the genistein diet was administered in the Santell et al. study. Therefore, different exposure time to genistein (before or after tumor formation) or interaction with a different tissue microenvironment might account for the different results.

More data are required to establish how genistein affects the growth of ER-negative breast cancer cells; however, genistein did not appear to promote the cell proliferation in ER-negative breast cancer cells. Moreover, molecular mechanisms by which genistein inhibits the proliferation of ER-negative breast cancer cells have not been suggested and need to be studied to support the observation of the tumor suppressive effect of genistein and to provide information that can be validated in human breast tumors.

## Effect of Daidzein Metabolite, Equol, on the Growth of Breast Cancer Cells

Daidzein, the second most prominent isoflavone found in soy, and soy products, has been shown to have much less biological effect compared to genistein in vitro (Peterson and Barnes 1991, 1996). However, daidzein can be further metabolized to equol by intestinal microflora (Setchell et al. 2005; Setchell and Clerici 2010b). Equol is structurally similar to estrogen and has a high affinity for ER*β* and, thus, has a potential to exert a greater estrogenic effect compared to the parent compound, daidzein (Setchell et al. 2005; Setchell and Clerici 2010b). However, only 30–50% people have intestinal bacteria that produce equol (Atkinson et al. 2008; Setchell and Clerici 2010a), while all rodents are equol producers (Setchell and Clerici 2010a,b). The effect of equol on the growth of breast cancer cells varied depending on the doses and cancer cell lines.

Equol enhanced cell proliferation of MCF-7 cells at concentrations of 1–10 *μ*mol/L (Ju et al. 2006; Choi and Kim 2008). However, dietary equol did not stimulate the growth of breast tumors (MCF-7 cells) implanted in ovariectomized mice at the doses of 250–1000 mg/kg equol that gave rise to the plasma concentration of about 3 *μ*mol/L (Ju et al. 2006). In another study, equol did not exert MCF-7 growth-promoting effect at any concentrations (1–100 *μ*mol/L) but significantly reduced the MCF-7 cell viability at concentrations higher than 75 *μ*mol/L (Charalambous et al. 2013). Thus, equol inhibited MCF-7 cell proliferation only at high concentrations, which may not be easily achieved by dietary intake and did not present an adverse effect in vivo.

The reported effect of equol on ER-negative breast tumors is inconsistent. Equol significantly inhibited proliferation of MDA-MB-453 cells in a dose- and time-dependent manner (Choi and Kim 2008). The growth suppression induced by high concentrations (50 and 100 *μ*mol/L) of equol was related to cell cycle arrest in the G_1_/S and G_2_/M phases as well as increase of expression of the proapoptotic protein (e.g., p53 and Bax) and decrease of the anti-apoptotic protein (e.g., Bcl-2) (Choi and Kim 2008). In contrast, equol increased cell proliferation of MDA-MB-435 cells and elevated both mRNA and protein expression of*c-myc* at concentrations of 5–50 *μ*mol/L (de la Parra et al. 2012). Equol also up-regulated eukaryotic protein synthesis initiation factors (eIFs) eIF4G, which may lead to the increase in protein expression related to cell survival and proliferation of MDA-MB-435 cells (de la Parra et al. 2012). It was speculated that equol may be involved in the elevation of eIF4G expression in MDA-MB-435 cell-derived tumors and increase of cell metastasis in female athymic mice treated with 10 mg/kg daidzein by oral gavage (Martinez-Montemayor et al. 2010; de la Parra et al. 2012). However, equol inhibited invasion of MDA-MB-231 cell at a concentration as low as 2.5 *μ*mol/L and down-regulated matrix metalloprotease-2, which promotes cell invasion (Magee et al. 2014). Moreover, the identity of MDA-MB-435, known as an ER-negative breast cancer cell line, has been questioned, and many believe that MDA-MB-435 cell line is derived from melanoma (Rae et al. 2007; Chambers 2009; Lacroix 2009). Therefore, more studies will be required to verify if the study results using MDA-MB-435 cell line that are relevant in the context of breast cancer.

Equol inhibited breast cancer cell growth only at high concentrations, close to 100 *μ*M, and the effect of equol on cell proliferation may not be significant in vivo except in MDA-MB-435 cells that has been suspected to be a melanoma cell line. Considering the high effective dose of equol and less than 50% equol producers in the human population, it is unlikely that equol consumption has a significant effect on human breast tumors.

## Combinational use of Isoflavones and Tamoxifen on Breast Cancer Growth

Tamoxifen has been used for treatment of ER*α*-positive breast tumors that requires estrogen to grow due to the antagonistic effect of hydroxytamoxifen (an active metabolite of tamoxifen) to the breast ER (Ring and Dowsett 2004). Since soy isoflavones can exert an estrogen agonistic/antagonistic effect, it was postulated that soy intake may enhance or interfere with anticancer activity of tamoxifen. Therefore, many studies evaluated the effect of combinational treatment of isoflavones and tamoxifen on the growth of ER-positive breast cancer cells.

### Combinational use in ER-positive breast cancers

Combinational treatment of tamoxifen and genistein inhibited the growth of ER-positive/HER2-overexpressing BT-474 human breast cancer cells in a synergistic manner (Mai et al. 2007). This synergistic growth inhibition was related to cell cycle arrest at G_1_ phase as well as induction of apoptosis with the maximum combination effect at 5 *μ*mol/L tamoxifen and 25 *μ*mol/L genistein (Mai et al. 2007). Co-treatment of tamoxifen and genistein inhibited protein expression of survivin (an inhibitor of apoptosis), EGFR family (EGFR, HER2, and HER3), and ER*α* in BT-474 cells compared to negligible inhibition by each agent (Mai et al. 2007). The high concentration of equol also synergistically inhibited the ER*α*-positive MCF-7 cell viability. The combination of equol (100 *μ*mol/L) and 4-hydroxy-tamoxifen (10 *μ*mol/L) induced caspase-mediated apoptosis more effectively than each compound alone (Charalambous et al. 2013). Therefore, high concentrations of genistein (>25 *μ*mol/L) or equol (100 *μ*mol/L) might be required to enhance tamoxifen efficacy on ER*α*-positive breast cancer.

In contrast, some studies have indicated that genistein impedes anticancer activity of tamoxifen in MCF-7 cells. Dietary genistein (1000 mg/kg) abrogated the tamoxifen-mediated growth inhibition of MCF-7 cells subcutaneously inoculated in ovariectomized athymic mice in the presence of a 0.25 mg estrogen implant (Ju et al. 2002). With a similar study design, except for the concentration of tamoxifen and estrogen (a 0.8 mg estrogen implant), the same group reported that lower doses of genistein (250, 500 mg/kg), but not a dose of 1000 mg/kg, negated the suppression of MCF-7 tumor growth by tamoxifen (Du et al. 2012). It was speculated that the different results with the 1000 mg/kg genistein diet (nullifying or no effect) on tamoxifen efficacy might be due to the difference in the ratio of estrogen to tamoxifen implants between the two studies (Du et al. 2012), making it difficult to predict genistein effect on breast cancer patients undergoing tamoxifen treatment. In an MCF-7 and H295R co-culture model where estrogen is supplied by H295R, genistein (1–10 *μ*mol/L) also reversed the growth inhibition induced by hydroxytamoxifen and further enhanced cell proliferation (van Duursen et al. 2013). Du et al. (2012) reported that genistein abrogated the growth inhibitory effect of tamoxifen only in the presence of estrogen. Therefore, a low level of genistein appeared to remove the therapeutic effect of tamoxifen on MCF-7 cells in the presence of estrogen.

The ratio of estrogen, genistein, and tamoxifen concentrations has been suggested to be critical in determining genistein effect on therapeutic efficacy of tamoxifen. However, it is difficult to predict the outcome of the interplay of those chemicals as there are many genetic and environmental factors that contribute to change in their metabolisms (Coughlin and Piper 1999; Jin et al. 2005). Furthermore, the relative ratio of estrogen to genistein concentrations in the serum does not reflect those of the breast tissue and tumor (Maubach et al. 2004; Yaghjyan and Colditz 2011). Therefore, studies need to be more carefully designed to consider concentrations of genistein, estrogen, and tamoxifen at the target site to estimate the effect of genistein on tamoxifen-treated ER-positive breast cancer. It should be also noted that tamoxifen acts in an ER-independent manner as well, although the anticancer activity of tamoxifen has been attributed to the induction of cell cycle arrest and apoptosis by inhibiting ER signaling (Weng et al. 2008). In addition, it will be important to expand findings to ER-positive cells other than MCF-7 line and identify molecular mechanisms that dictate the role of genistein in tamoxifen efficacy in order to verify them in the human disease.

### Combinational use in ER-negative breast cancers

In ER*α*-negative MDA-MB-231 breast cancer cells, genistein seemed to act favorably on tamoxifen efficacy, although there are insufficient studies to derive any firm conclusions. A study suggested that genistein sensitized tamoxifen effect in ER*α*-negative breast cancer cells through its epigenetic regulation. Genistein reactivated*ERα* expression through the histone modification in the*ERα* promoter and the inhibition of histone deacetylase inhibitors in MDA-MB-231 and MDA-MB-157 cells (Li et al. 2013). Dietary genistein (250 mg/kg) also restored*ERα* transcription, probably through the inhibition of expression and activity of enzymes that regulate chromatin structure in MDA-MB-231 cell-derived tumors, and significantly increased the anticancer efficacy of tamoxifen (Li et al. 2013). Genistein (2 *μ*mol/L) elevated both mRNA and protein expression of phosphatase and tensin homolog (PTEN) in the ER-negative nontumorigenic human mammary epithelial cells, MCF-10A (Rahal and Simmen 2010). However, this study focused on genistein-induced cross-regulation of PTEN and p53, but not genistein-derived epigenetic modification of PTEN. Epigenetic regulation by equol was reported as well. Equol treatment (2 *μ*mol/L) significantly decreased the DNA methylation of the CpG islands in the promoters of tumor suppressor genes,*BRCA1* and*BRCA2*, in both MDA-MB-231 and MCF-7 cells, subsequently increasing their expressions in both transcription and protein levels (Bosviel et al. 2012). However, this study did not clearly elucidate the mechanism for equol-induced demethyation. Thus, these studies suggest that epigenetic modulation by genistein and equol might contribute to their anticancer activity in breast cancer.

ER-negative tumors do not respond to tamoxifen treatment and more aggressive, resulting in a poorer prognosis (Rochefort et al. 2003). Therefore, tamoxifen and genistein combination therapy might be a good option for ER-negative breast cancer and needs to be studied further. However, questions still remain as to whether breast tumors that restore ER and respond to tamoxifen by genistein treatment will then be refractory to tamoxifen due to the abrogation of the effect of tamoxifen by genistein later. In addition, it should be further evaluated whether genistein can accumulate enough to exert biological activity in human tumors due to low bioavailability of isoflavones in humans (Setchell et al. 2011). Nevertheless, it is noteworthy that genistein or equol can enhance the expression of tumor suppressor genes such as*BRCA1*,*BRCA2*, and*PTEN* at concentrations as low as 2 *μ*mol/L in both ER-positive and -negative breast cancer cells and should be further elucidated as an anticancer mechanism of soy isoflavones in human breast cancer.

## Conclusion

Based on available preclinical data, the high concentration of genistein and equol, which might be difficult to be achieved in human breast tissues or tumors, effectively inhibited the growth of breast cancer cells regardless of their ER status. However, genistein at low concentration oppositely stimulated ER-positive breast tumors and abrogated tamoxifen-mediated growth inhibition in ER-positive breast cancer. A potential estrogenic effect of genistein was suggested as the mechanism by which genistein enhances estrogen-dependent tumor growth. Hence, the relative levels of genistein, estrogen, and tamoxifen at the target site are important in determining genistein effect on the growth of ER-positive breast tumors. However, estrogen concentration might be underestimated in preclinical studies that utilize ovariectomized mice and subcutaneous xenografts models. Estrogen concentration is high in human breast tissues and even higher in breast tumors compared to serum. Besides, recent studies demonstrated nonestrogenic anticancer effect of isoflavones (e.g., reactivation of epigenetically silenced tumor suppressor genes) and ER-independent mechanism of anticancer activity of tamoxifen. Therefore, it may be oversimplifying to assume that isoflavones promote ER-positive breast cancer cells and abolish tamoxifen anticancer activity due to their potential estrogenic effect.

In future preclinical studies, the relative levels of isoflavones and estrogen at the target site should be considered more carefully in the study design, and different ER-positive breast cancer cells other than MCF-7 need to be utilized to generalize preclinical findings. In addition, parameters that can estimate relative levels of estrogen and isoflavones should be included to determine the effect of isoflavones on the growth of human breast tumors in clinical/epidemiological studies. It is also critical to identify the molecular mechanisms by which soy isoflavones exert cancer promotion or inhibition effect to validate these in the human disease and to use them as the molecular markers that could predict isoflavones effects on human breast tumor.
